# Assessing Reactive and Proactive Aggression in Detained Adolescents Outside of a Research Context

**DOI:** 10.1007/s10578-015-0553-z

**Published:** 2015-06-02

**Authors:** Olivier F. Colins

**Affiliations:** Department of Child and Adolescent Psychiatry, Curium-Leiden University Medical Center, Endegeesterstraatweg 27, 2342 AK Oegstgeest, The Netherlands; Academic Workplace Forensic Care for Youth (Academische Werkplaats Forensische Zorg voor Jeugd), Zutphen, The Netherlands

**Keywords:** Aggression, Detained, Cluster-analyses, Adolescents, Ethnicity, Validation, Proactive, Reactive

## Abstract

The Reactive Proactive Aggression Questionnaire (RPQ) is a self-report tool for assessing reactive aggression (RA) and proactive aggression (PA). This study contributes to the literature by testing the psychometric properties of the RPQ across detained boys from various ethnicities whilst using data that were gathered during clinical assessments. The factorial, convergent, and criterion validity, and the internal consistency of the RPQ scores received strong support in the total sample and across four ethnicity groups. Also, three groups of boys were identified, with the group including boys with high levels of both RA and PA including the most severe boys in terms of anger, delinquency, alcohol/drug use, and psychopathic traits, and having the highest prevalence rate of conduct disorder and substance use disorder. Together, these findings suggest that the RPQ may hold promise for assessing RA and PA in detained boys, even when confidentiality and anonymity of the information is not guaranteed.

**Electronic supplementary material:**

The online version of this article (doi:10.1007/s10578-015-0553-z) contains supplementary material, which is available to authorized users.

## Introduction

Aggression is an umbrella term that captures different types of aggression that differ in underlying mechanisms, forms, functions, and prognosis [1]. One distinction that received a lot of attention relates to the differentiation between proactive and reactive aggression. Reactive aggression is a hostile, impulsive, and unplanned reaction to perceived frustration or threat [2]. Reactive aggression is often accompanied by anger and rage, autonomic arousal and loss of impulse control, and, therefore, has been described as a ‘hot’ form of aggression [3, 4]. Proactive aggression is displayed in the absence of provocation or anger [5], with the goal to take possession of things, or to dominate or intimidate others [6]. Because no or only little autonomic arousal is involved, proactive aggression has been described as a ‘cold’ form of aggression [3]. Although reactive aggression and proactive aggression are highly correlated [7], previous research in adolescents has shown that both types of aggression differ in the direction and/or strength of relations to variables of interest. With respect to internalizing problems, reactive aggression is positively related to depression [8], suicide risk [9], and anxiety [10], whereas proactive aggression is not. With respect to externalizing problems, proactive aggression is positively correlated to conduct problems [8], bullying [11] and substance abuse [7], whereas reactive aggression is not, or less strongly, related to these features. In addition, reactive aggression, but not proactive aggression, is positively related to impulsivity [12]. With respect to offending, proactive aggression, but not reactive aggression, is independently and positively related to offending in general [12], and violent offending in specific [7], though it must be noted that studies have also shown a positive relationship between reactive aggression and offending, or did not reveal any relationship between both types of aggression and offending [13]. With regard to psychopathic traits, studies have shown that these traits were merely or most consistently related to proactive aggression [14, 15].

The Reactive Proactive Aggression Questionnaire (RPQ; 12) is a promising self-report questionnaire designed to assess reactive and proactive aggression in children and adolescents (see “Methods” for more details). Factor analyses supported the two-factor structure of the RPQ in different settings [11, 14], in different countries/cultures [16, 17], and in boys and girls [16]. Overall, the internal consistencies of the RPQ total score and the reactive aggression (RA) and proactive aggression (PA) scale scores are acceptable to very good [14, 18]. Finally, the convergent and criterion validity of RPQ scores are supported by evidence that the total score, and the RA and PA scales manifested the expected relationships with variables of interest [8, 12, 14]. As parents or teachers of detained adolescents are very often not available, unable or unwilling to provide information [19, 20], the availability of a reliable and valid self-report tool is very much welcomed to assess reactive and proactive aggression among these criminal justice-involved adolescents. However, in clinical practice, detained adolescents may be reluctant to provide information that is unknown to their parents and clinicians (e.g., aggression, drug use), and can be used against them (e.g., in court). Therefore, it is highly relevant to test if RPQ scores are still as reliable and valid when detained youths complete the RPQ as part of a clinical protocol, thus, outside of a research context where anonymity and confidentiality of the information is guaranteed.

## This Study: Aims and Hypotheses

Worldwide, youths who are culturally different from the culture of the host nation are overrepresented in youth detention centers [21]. However, we are aware of no study that has examined the psychometric properties of the RPQ across detained youths from various ethnicities. As such, the first aim of the present study was to fill this void by examining the factorial, convergent and criterion validity of RPQ scores. It was hypothesized that a good model fit for the two-factor structure and good internal consistencies of the RPQ scores would be revealed, not only in the total sample but also in the different ethnicity groups. In support of the convergent validity of RPQ scores, it was hypothesized that positive relationships between the RPQ scores and other indices of aggression would be revealed. Specifically, it was expected that the RA score would be strongly related to anger and irritability. In contrast, and because proactive aggression is often displayed in the absence of anger, it was expected that the relationship between the PA score and anger would be poor at best. In support of the criterion validity of RPQ scores, it was expected that the RA score would be most strongly and consistently positively related to depressive feelings, anxiety, and suicide risk, and that the PA score would be most strongly and consistently positively related to substance use, callous–unemotional traits, and offending.

Demonstrating different correlates for reactive and proactive aggression is important but not sufficient to support the usefulness of the dichotomy in clinical practice, in which clinicians deal with persons rather than variables. The second aim of this study was to test whether meaningful groups of detained youths could be identified that differ in their levels of reactive and proactive aggression. Prior work showed that individuals with proactive aggression typically show significant levels of reactive aggression as well [22]. In addition, several studies also identified a group of individuals with high levels of reactive aggression only and a group with low levels of proactive and reactive aggression (for an overview see [23]). Though not impossible, prior studies suggested that it is difficult to identify individuals that merely display high levels of proactive aggression [5, 23–25]. Interestingly, there is evidence that youths with high levels of reactive and proactive aggression differ from youths with reactive aggression only; with the former group showing the highest levels of aggression, impulsivity, bullying, anger dysregulation, and callous–unemotional traits [18, 22, 24]. Of note, these two groups were not different with respect to social competence, depressive–anxious feelings, expectation for rewards, and thrill-seeking behavior [18, 22, 24]. On the basis of prior research, it was hypothesized that detained adolescents with high scores on reactive and proactive aggression, and detained adolescents with high scores on reactive aggression only would be identified and that both groups would have more internalizing and externalizing problems, alcohol and drug use problems, and higher prevalence rates of conduct disorder and substance use disorder than youths with low scores of reactive and proactive aggression. In line with a severity model [22], youths with high scores on reactive and proactive aggression were also expected to have more problems and higher prevalence rates of conduct disorder and substance use disorder than youths with high scores on reactive aggression only.

## Methods

### Participants

The current study used data involving male adolescents from two large youth detention centers in the Netherlands. These data were gathered as part of the standardized mental health screening and assessment the two youth detention centers provide to each youth entering the institution. In the first phase of implementing standardized mental health screening and assessment (May 2008–July 2009), the Massachusetts Youth Screening Instrument-Second Version [26] and Strengths and Difficulties Questionnaire [27] were implemented. In the second phase (July 2009–July 2011), an extensive screening and comprehensive psychiatric assessment was implemented by means of self-report questionnaires (e.g., RPQ and self-reported offending), and structured psychiatric diagnostic interviews (see “Measures”). In the third phase (from July 2011), the presence of oppositional defiant disorder, conduct disorder, and substance use disorders other than alcohol and marijuana, was no longer assessed, and some self-report questionnaires were no longer administered to the youths (e.g., self-reported offending). For the purpose of the current study, data for 807 detained male adolescents who completed the RPQ, the Massachusetts Youth Screening Instrument-Second Version (MAYSI-2), and the Strengths and Difficulties Questionnaire (SDQ) were made available to the author. For a sub-sample of these 807 youths, data from other screening and assessment tools were available and were also used in the current study. Because of the developments described above (see the third phase) and because some disorders could not be assessed in some youths (e.g., release before the clinical screening and assessment protocol was complete), the number of youths that were used when examining the relationship between RPQ scores and variables of interest assessed by other instruments than the MAYSI-2 and SDQ will be lower than 807 (see “Measures”). The mean age of our sample (*N* = 807) was 16.71 years (*SD* = 1.30). With respect to ethnicity, 20.7 % were of Dutch ethnicity, 26.9 % were of Moroccan ethnicity, 19.8 % were of Dutch Antillean or Surinamese ethnicity, and 31.1 % were from another ethnicity (e.g., Turkish). Data regarding ethnicity were missing for 1.5 % of the sample. In addition, 95 % of the boys were detained while awaiting final trial (pretrial), whereas the remaining 5 % were detained following conviction. The number of days between entrance into the facility and completing the RPQ ranged from 0 to 66 days and was on average 5.66 (*SD* = 4.54).

### Materials

#### Reactive Proactive Aggression Questionnaire (RPQ)

The RPQ [12, 14] includes 23 items that were based on previous teacher-rating measures of reactive aggression and proactive aggression, and on conceptual and theoretical relevance. These items were developed to reflect physical or verbal aggression and the motivation and situational context for the aggression. Eleven items focus on reactive aggression (e.g., Reacted angrily when provoked by others, Gotten angry when frustrated), and twelve items focus on proactive aggression (e.g., Had fights with others to show who was on top; Taken things from other students). The items must be answered as never, sometimes or often.

#### Massachusetts Youth Screening Instrument-Second Version (MAYSI-2)

The MAYSI-2 [26, 28] is a 52-item screening tool (yes or no responses) in which youths report the presence or absence of symptoms or behaviors related to several areas of emotional, behavioral, and psychological disturbances experienced “within the past few months.” Factor analyses indicated that the items produce scores on six clinical scales: alcohol–drug use, angry–irritable, depressed–anxious, somatic complaints, suicide ideation, and thought disturbance (for boys only) and one non-clinical scale (traumatic experiences) that screens for exposure to potentially traumatic events. There is no MAYSI-2 total score as the developers did not intend to develop scales that contribute to a broader construct, such as internalizing problems [26]. For the purpose of this study, four scales of the MAYSI-2 were used: alcohol/drug use (eight items; α in the present study = .83); angry–irritable (nine items; α = .77); depressed–anxious (nine items; α = .66); and suicide ideation (five items; α = .73). The Dutch MAYSI-2 has been shown to have promising psychometric properties [29].

#### Strengths and Difficulties Questionnaire (SDQ)

The self-report version of the SDQ [27, 30] is a screening instrument for psychosocial functioning for children and adolescents. The SDQ has four difficulty subscales (hyperactivity, conduct problems, peer problems, emotional symptoms) and one strength subscale (prosocial behavior). Each of these five SDQ subscales consists of five items that need to be answered as being not true, somewhat true, and certainly true. In this study only the prosocial behavior subscale (five items; e.g., I often volunteer to help others; α = .62) was used.^1^ A high score on this scale is indicative of high levels of prosocial behavior.

#### Youth Self-Report (YSR)

The YSR [31] includes 118 ‘problem’ items that youths answer as being not true, sometimes true, or very true for themselves. The responses to the problem items contribute to eight narrow-band scales that identify problem areas (withdrawn/depressed, somatic complaints, anxious/depressed, social problems, thought problems, attention problems, rule-breaking behavior and aggressive behavior). For the purpose of this study, only the aggression behavior (17 items; α = .84) and social problems (11 items; α = .63) subscales were used. Data were available for 443 youths.

#### Diagnostic-Interview Schedule for Children-Fourth Version (DISC-IV)

The DISC-IV is a structured diagnostic interview that covers many psychiatric diagnoses in DSM-IV and ICD-10, and can be administered by trained non-clinicians [32, 33]. For the purpose of this study, the past year prevalence of conduct disorder (CD) and substance use disorder (SUD) was assessed. SUD refers to the presence of alcohol use disorder, marijuana use disorder, and/or other SUD (e.g., cocaine, amphetamines). In addition, a continuous variable was created, reflecting the number of aggressive DSM-IV CD symptoms that were reported (range 0–7). Finally, participants with CD who reported at least one aggressive CD symptom were also referred to as having aggressive CD. Data regarding CD and SUD were available for 424 and 537 youths, respectively.

#### Youth Psychopathic Traits Inventory (YPI)

The YPI [34] is a self-report questionnaire with 50 items that are organized into 10 subscales (with five items each) and three dimensions, including an interpersonal (α = .89), affective or callous–unemotional (α = .77), and behavioral/lifestyle dimensions (α = .86). Each item in the YPI is scored on a four-point Likert scale, ranging from “Does not apply at all” to “Applies very well.” Data were available for 757 youths.

#### Self-Reported Offending

The Research and Documentation Center Monitor [35] was used to determine whether or not participants ever committed an offense. Based on previous studies [36], five continuous offense categories were created. First, seven items referring to violence were classified as “violent offenses” (e.g., using violence to steal from someone, trying to have sex while the other refuses). Second, 11 items referring to income-related non-violent delinquent behaviors were classified as “property offenses” (e.g., burglary, shoplifting, theft in school). Third, five items referring to deliberately damaging property were classified as “vandalism” (e.g., damaging a car). Fourth, three items referring to dealing or selling drugs were classified as “drug-related offenses” (e.g., selling hard drugs). Fifth, five items referring to threatening and insulting were classified as “threats and insults” (e.g., threatening someone to make him or her scared, insulting someone because the other person is homosexual). Data were available for 430 (violent offenses) up to 438 (drug-related offenses) youths.

#### Ethnicity

Based on the Dutch standard classification of ethnicity, participants were categorized as “Moroccan” or “Antillean/Surinamese” when the adolescent and/or at least one parent had been born in Morocco, or the Dutch Antilles or Surinam, respectively. When both parents were of non-Dutch ethnicities, we used the country of birth of the mother to determine the ethnicity of the child. Participants were classified as Dutch when both parents and the child were born in The Netherlands. All other participants (e.g., from Turkish, Afghan, Italian, and Polish origin) were assigned to the mixed ethnicity group.

### Procedure

The MAYSI-2 and SDQ were administered on a stand-alone computer in the presence of non-clinical personnel from the youth detention centers (YDCs) to all youths within a few days after detention entry. Master students and test assistants with a Master’s degree trained by clinically experienced researchers performed the comprehensive assessments. Youths were aware that the mental health screening and assessment were part of the YDCs’ clinical protocol, and that the outcomes from mental health screening and assessment were available to YDCs personnel. Through standardized information provided by the YDCs upon start of detention, youths and their parents/care-takers were informed that the mental health screening and assessment outcomes would be used for scientific research, unless they declined (cf. passive informed consent). They were also informed that, if they did not decline, their information would be transferred anonymously to the researchers, so that it would be impossible to trace the information back to them. Given that routine mental health screening and assessment was part of clinical care, the relevant boards of the YDCs waived the requirement to obtain active informed consent from youths, and for youths <18 years of age, to obtain active informed consent from their parent(s)/caretaker(s) as well. The Medical Ethical Review Board of the Leiden University Medical Center certified that the study met the Dutch law of behavioral research because all data were derived as part of the clinical assessment.

### Data-Analyses

To test the two-factor model of the RPQ, confirmatory factor analyses (CFA) were performed using Mplus 6.1 [37]. Because the items of the RPQ are scored on an ordinal scale, the robust weighted least squares estimator was used. Model fit was assessed using *χ*^2^, root mean square error of approximation (RMSEA), and the comparative fit index (CFI). RMSEA scores <.05 indicated a good fit, whereas scores between .05 and .08 indicated an acceptable fit. A CFI score ≥.95 indicated an excellent fit, and a CFI score ≥.90 indicated a good fit [38]. With respect to *χ*^2^, a good fit is indicated when *χ*^2^/*df* ≤ 2, whereas *χ*^2^/*df* ≤ 3 is indicative of an acceptable fit [39]. To evaluate the internal consistency of the RPQ scores, Cronbach’s alphas (α) were calculated. Reliability coefficients were interpreted as follows: <.60 = insufficient; .60–.69 = marginal; .70–.79 = acceptable; .80–.89 = good, and .90 or higher = excellent [40]. Differences between ethnicity subgroups were examined using one-way analyses of variance for continuous variables (e.g., RPQ scores) and Chi square tests for categorical variables (e.g., CD) using Bonferroni correction for multiple comparisons. In case assumptions for one-way analyses of variance were violated differences between ethnic subgroups were examined with a series of Mann–Whitney tests (*U*). To test the convergent and criterion validity of the RPQ total score and the RA and PA scores, bivariate linear regression analyses (in case of continuous dependent variables) and bivariate logistic regression analyses (in case of categorical dependent variables) were performed. To test the unique association between the RA and PA scores on the one hand and variables of interest on the other hand, these regression analyses were repeated while simultaneously including the RA and PA scores as a predictor. Of note, the RPQ total, the RA, and PA scores were not significantly related to age in the total sample or in the four ethnicity groups, so age was not included in the regression analyses. K-means cluster analyses were performed to test whether meaningful clusters of youths could be identified that differ in standardized RA and PA scores. Between cluster-comparisons were performed in the same way as described for the between ethnicity groups comparison. Because of the large number of significance tests conducted, an alpha of *p* < .01 was used as an indicator for statistical significance. All analyses were performed using SPSS 20.0 unless otherwise specified.

## Results

### Variable-Oriented Analyses

#### Factorial Validity and Internal Consistency

Model fit indices of the two-factor model for the total sample were in the adequate range, with the exception of the *χ*^2^/*df* ratio, which was 4.17, and thus indicative of an unacceptable fit (Table 1). For the four ethnicity groups, the *χ*^2^/*df* ratio and RMSEA were below (i.e., Moroccan and mixed) or just above (i.e., Dutch and Antillean/Surinamese) the recommended cut-off values, and thus indicative of a good model fit. Also, in all but one of the ethnicity groups, the CFI was greater than the .95 CFI cut-off value, and thus also indicative of a good model fit. For Antillean/Surinamese youths, the CFI suggested the model fit to be acceptable (.93). The relative *χ*^2^ difference test indicated a significantly better fit for the two-factor model over the one-factor model in the total sample (*χ*^2^ = 26.68, *df* = 1, *p* < .001) and in all four ethnicity groups (Dutch: *χ*^2^ = 32.43, *df* = 1, *p* < .001; Moroccan: χ^2^ = 26.68, *df* = 1, *p* < .001; Antillean/Surinamese: *χ*^2^ = 21.44, *df* = 1, *p* < .001; mixed ethnicity: *χ*^2^ = 33.00, *df* = 1, *p* < .001). Table 1 also shows good-to-excellent internal consistency of the RPQ total score, and the RA and PA scores. In Antillean/Surinamese youths, α for the PA scale was just below the cut-off value for good reliability, but was still acceptable. Finally, the correlation between the RA and PA scores was .66 (total sample), .65 (Dutch), .61 (Moroccan), .64 (Antillean/Surinamese), and .62 (mixed ethnicity). All correlations were significant at *p* < .001.

**Table 1:** Model fit indices for the two factor model (proactive–reactive aggression) and the one factor model (general aggression) and reliability indices for the RPQ total score, RPQ reactive and RPQ proactive subscales

	Two factor model				One factor model				Cronbach’s α		
	*χ* ^2^	*df*	RMSEA	CFI	*χ* ^2^	*df*	RMSEA	CFI	Total	RA	PA
Total sample (*N* = 794)	955.70	229	.063	.931	982.33	230	.064	.928	.90	.85	.83
Dutch sample (*N* = 164)	336.85	229	.054	.950	398.44	230	.067	.922	.90	.85	.83
Moroccan sample (*N* = 215)	294.83	229	.037	.975	335.17	230	.046	.960	.88	.84	.81
Antillean/Surinamese sample (*N* = 156)	336.40	229	.055	.932	370.16	230	.063	.911	.88	.83	.79
Mixed ethnicity sample (*N* = 247)	350.39	229	.046	.958	420.70	230	.058	.934	.89	.84	.82

For 13 out of the 807 boys there were one or two missing RPQ item scores. These boys were not included in the factor analyses, but were included in all other analyses; For 12 boys information about ethnic origin were missing. These boys were not included in the factor analyses within the ethnic groups, but were included in all other analyses

*CFI* comparative fit index, *RMSEA* root mean square error of approximation, *RA* reactive aggression, *PA* proactive aggression

#### RPQ Mean Scores

Table 2 shows the mean RPQ scores for the total sample and the four ethnicity groups. Moroccan boys had significantly lower RPQ total and RA and PA scores than all three other ethnicity groups, and boys of mixed ethnicity had significantly lower scores than Dutch and Antillean/Surinamese youths. The magnitude of the differences between Moroccan and Dutch boys was moderate (Cohen’s *d*: total = .71; RA = .66; PA = .61), whereas the magnitude of the differences between Moroccan and Antillean/Surinamese boys ranged from moderate-to-almost large (Cohen’s *d*: total = .79; RA = .75; PA = .66). *D*’s for significant group differences presented in Table 2 but not described here were (far) below .50 (available upon request).

**Table 2:** Mean (M) scores (SD) on the Reactive Proactive Aggression Questionnaire and differences between groups of youth from various origin

	Total sample (*N* = 807)		Dutch (1) (*N* = 167)		Moroccan (2) (*N* = 217)		Antil/Surin (3) (*N* = 160)		Mixed (4) (*N* = 251)		Group comparison^a^
	*M*	*SD*	*M*	*SD*	*M*	*SD*	*M*	*SD*	*M*	*SD*	
Total score	9.94	6.87	11.87	7.22	7.19	5.79	12.20	6.78	9.53	6.53	2 < 1, 3, 4; 4 < 1, 3
Reactive aggression	7.38	4.39	8.54	4.48	5.70	4.02	8.82	4.26	7.11	4.16	2 < 1, 3, 4; 4 < 1, 3
Proactive aggression	2.56	3.12	3.33	3.46	1.49	2.38	3.37	3.21	2.41	3.07	2 < 1, 3, 4; 4 < 1, 3

The sum of the number of participants in each subsample does not equal 807 due to missing information about ethnicity for 12 participants Antil/Surin = Antillean/Surinamese

^a^Based on Mann–Whitney (*p* < .01)

#### Convergent Validity

In the total sample, the RPQ total score was significantly positively related to aggressive behavior, angry–irritability and number of aggressive CD symptoms (Table 3). At the zero-order level, both RPQ scale scores were positively related to these outcomes as well. After controlling for the PA score, the RA score remained significantly related to angry–irritable features and aggressive behavior, but was no longer significantly related to the number of aggressive CD symptoms. After controlling for the RA score, the PA score remained significantly related to aggressive behavior, aggressive CD symptoms and angry–irritability (Table 3).

**Table 3:** Convergent validity of the Reactive Proactive Aggression Questionnaire for the total sample and for youths from various origin

	Total sample			Dutch			Moroccan			Antillean/Surinamese			Mixed ethnicity		
	Aggress.	CD sym.	Anger	Aggress.	CD sym.	Anger	Aggress.	CD sym.	Anger	Aggress.	CD sym.	Anger	Aggress.	CD sym.	Anger
Total	.73**	.41**	.60**	.75**	.46**	.56**	.72**	.47**	.60**	.71**	.31*	.66**	.74**	.32*	.54**
Reactive (zero-order)	.69**	.33**	.60**	.71**	.33**	.57**	.67**	.32**	.64**	.66**	.28*	.64**	.70**	.27*	.53**
Reactive (adjusted)	.46**	.03	.51**	.49**	−.11	.49**	.52**	.02	.62**	.32*	.10	.50**	.48**	.12	.42**
Proactive (zero-order)	.65**	.44**	.47**	.66**	.54**	.44**	.57**	.57**	.42**	.69**	.31**	.54**	.64**	.31*	.43**
Proactive (adjusted)	.32**	.43**	.14**	.32*	.62**	.12	.30**	.56**	.07	.44**	.23	.22*	.33**	.23	.17

*Aggress.* youth self-report aggressive behavior, *CD symp.* number of aggressive conduct disorder symptoms, *Anger* MAYSI-2 angry-irritable

* *p* < .01; ** *p* < .001

In each of the four ethnicity groups,^2^ results were generally similar to the results reported for the total sample, with two exceptions, though. First, after controlling for the RA score, the PA score was not significantly related to aggressive CD symptoms Antillean/Surinamese and mixed ethnicity boys. Second, after controlling for the RA score, the PA score was not significantly related to the angry–irritability in boys from Dutch, Moroccan and mixed ethnicity (Table 3).

#### Criterion Validity

In the total sample, all RPQ scores (zero-order level) were negatively related to prosocial behavior and positively related to all other outcomes presented in Table 4. After controlling for the PA score, the RA score was positively related to symptoms of depression/anxiety, suicide ideations, social problems, alcohol and drug use, SUD, psychopathic traits and threats/insults, but unrelated to prosocial behavior, (aggressive) CD, violent offenses, theft, vandalism and drug offenses. After controlling for the RA score, the PA score was not significantly related to symptoms of depression/anxiety, suicide ideations, social problems, negatively related to prosocial behavior, and positively related to all the other outcomes.

**Table 4:** Criterion validity of the Reactive Proactive Aggression Questionnaire (total sample)

	Depressed/anxious	Suicide ideation	Social problems	Prosocial behavior	Alcohol/drug use	Substance use disorder	CD	CD aggressive	YPI total score
Total	.33**	.20**	.38**	−.28**	.48**	1.17**	1.18**	1.17**	.69**
RA (zero-order)	.34**	.21**	.37**	−.22**	.42**	1.24**	1.25**	1.23**	.61**
RA (adjusted)	.32**	.21**	.29**	−.05	.18*	1.13**	1.03	1.01	.33**
PA (zero-order)	.24**	.14**	.28**	−.30**	.48**	1.39**	1.45**	1.39**	.65**
PA (adjusted)	.03	<.01	.10	−.26**	.36**	1.13**	1.41**	1.37**	.44**

	YPI ID	YPI AD	YPI BD	Violent offenses	Theft	Vandalism	Threats/insults	Drug offenses	
Total	.54**	.50**	.66**	.60**	.75**	.59**	.62**	.46**	
RA (zero-order)	.46**	.42**	.63**	.50**	.46**	.48**	.53**	.36**	
RA (adjusted)	.18**	.15*	.46**	.11	.07	.08	.15*	.01	
PA (zero-order)	.54**	.51**	.56**	.63**	.61**	.63**	.64**	.51**	
PA (adjusted)	.43**	.42**	.26**	.56**	.57**	.57**	.54**	.51**	

*CD* conduct disorder, *YPI* youth psychopathic traits inventory, *ID* interpersonal dimension, *AD* affective dimension, *BD* behavioral dimension, *RA* reactive aggression, *PA* proactive aggression, *Surin.* surinamese

* *p* < .01; ** *p* < .001

When repeating the analyses in each of the four ethnicity groups (Table 5), the pattern of relationships between the RPQ scores (zero-order level) and variables of interest were substantially the same as reported for the total sample. Table 5 also shows that after controlling for the other score the pattern of relationships between the RA or PA scores and variables of interest were mainly the same as reported for the total sample, with a few notable exceptions being described next. After controlling for PA, RA was not related to feelings of depression and anxiety in boys from mixed ethnicity, not related to suicide ideations in boys from Dutch and mixed ethnicity; not related to social problems in Dutch and Antillean/Surinamese boys; and not related to alcohol/drug use, substance use disorder, the interpersonal and affective psychopathy dimensions, and threats/insults in Dutch, Moroccan, and Antillean/Surinamese boys. After controlling for RA, PA was not related to prosocial behavior in Dutch and mixed ethnicity boys, and was not related to SUD, aggressive CD, and threats/insult in boys from mixed ethnicity.

**Table 5:** Criterion validity of the Reactive Proactive Aggression Questionnaire in ethnic groups

	Depressed/anxious	Suicide ideation	Social problems	Prosocial behavior	Alcohol/drug use	SUD	CD	Aggressive CD	YPI total
Dutch									
Total	.32**	.06	.30*	−.24*	.52**	1.14**	1.21**	1.16**	.60**
RA (zero-order)	.30**	.10	.25*	−.19	.40**	1.17**	1.25**	1.18*	.50**
RA (adjusted)	.20**	.20	.07	−.06	.07	1.02	.92	.84	.19
PA (zero-order)	.27**	−.05	.31*	−.25*	.56**	1.44**	1.66**	1.47**	.60**
PA (adjusted)	.13	−.12	.26	−.21	.51**	1.40*	1.80**	1.75**	.48**
Moroccan									
Total	.38*	.17	.34**	−.29**	.42**	1.19**	1.36**	1.35**	.70**
RA (zero-order)	.44*	.21*	.37**	−.20*	.35**	1.24**	1.31*	1.32*	.61**
RA (adjusted)	.51**	.28*	.38**	.05	.15	1.11	1.08	1.14	.33**
PA (zero-order)	.19*	.06	.19	−.38**	.42**	1.44**	2.16**	1.82**	.66**
PA (adjusted)	−.19	−.11	−.01	−.41**	.33**	1.32*	2.05**	1.71**	.46**
Antillean/Surin.									
Total	.39**	.33**	.36**	−.19	.46**	1.14**	1.21**	1.16**	.62**
RA (zero-order)	.41**	.34**	.36**	−.10	.42**	1.17*	1.25**	1.18*	.50**
RA (adjusted)	.37**	.31*	.29	.13	.24	1.02	.92	.10	.19
PA (zero-order)	.29**	.25*	.31*	−.27**	.43**	1.44**	1.66**	1.47**	.64**
PA (adjusted)	.05	.05	.09	−.36**	.29*	1.40*	1.80**	1.75**	.53**
Mixed ethnicity									
Total	.23**	.21*	.39**	−.24**	.45**	1.16**	1.13*	1.13*	.76**
RA (zero-order)	.21*	.17*	.40**	−.18	.40**	1.26**	1.23*	1.23*	.70**
RA (adjusted)	.15	.10	.36*	−.10	.22*	1.20*	1.17	1.16	.47**
PA (zero-order)	.19*	.21*	.29*	−.21*	.42**	1.27**	1.22*	1.23*	.66**
PA (adjusted)	.10	.17	.06	−.10	.28**	1.10	1.10*	1.11	.38**

	YPI ID	YPI AD	YPI BD	Violent offenses	Theft	Vandalism	Threats/insults	Drug offenses
Dutch								
Total	.47**	.38**	.58**	.63**	.64**	.62**	.59**	.48**
RA (zero-order)	.38**	.28**	.54**	.52**	.49**	.48**	.42**	.37**
RA (adjusted)	.09	−.13	.35**	.10	−.03	−.01	−.13	−.03
PA (zero-order)	.50**	.43**	.52**	.66**	.71**	.68**	.69**	.54**
PA (adjusted)	.43**	.44**	.29*	.60**	.73**	.69**	.79**	.56**
Moroccan								
Total	.53**	.51**	.71**	.39**	.53**	.48**	.62**	.44**
RA (zero-order)	.44**	.42**	.66**	.28*	.38**	.32**	.45**	.34**
RA (adjusted)	.17	.17	.48**	.07	.09	.00	.07	.10
PA (zero-order)	.55**	.52**	.60**	.46**	.61**	.60**	.72**	.49**
PA (adjusted)	.45**	.42**	.31**	.42**	.56**	.60**	.68**	.44**
Antillean/Surin.								
Total	.47**	.44**	.61**	.65**	.49**	.54**	.58**	.46**
RA (zero-order)	.36**	.32**	.54**	.52**	.41**	.47**	.51**	.35*
RA (adjusted)	.08	.02	.34**	−.05	.03	.11	.13	−.10
PA (zero-order)	.51**	.51**	.55**	.72**	.53**	.56**	.60**	.53**
PA (adjusted)	.46**	.50**	.34**	.75**	.51**	.47*	.50**	.60**
Mixed ethnicity								
Total	.59**	.62**	.66**	.60**	.61**	.60**	.66**	.45**
RA (zero-order)	.51**	.54**	.66**	.51**	.51**	.51**	.64**	.36**
RA (adjusted)	.28**	.31**	.54**	.23	.21	.20	.50**	.07
PA (zero-order)	.56**	.57**	.52**	.58**	.68**	.60**	.55**	.49**
PA (adjusted)	.38**	.38**	.19*	.43**	.47**	.46**	.23	.44**

*SUD* substance use disorder, *CD* conduct disorder, *YPI* youth psychopathic traits inventory, *ID* interpersonal dimension, *AD* affective dimension, *BD* behavioral dimension, *RA* reactive aggression, *PA* proactive aggression, *Surin.* Surinamese

* *p* < .01; ** *p* < .001

### Person-Oriented Analyses

#### Deriving Clusters

K-means cluster analyses were performed to determine whether three clusters of youths could be meaningfully identified in the total sample. The results showed a cluster with boys scoring >½ *SD* below the mean on RA and PA (labeled as low aggression), a cluster with boys scoring >1 *SD* above the mean on RA and PA (labeled as combined aggression), and a cluster of boys scoring >½ *SD* above the mean for RA and close to the mean for PA (labeled as reactive aggression only). Next, it was tested whether a four-cluster solution would identify a group of youths with high scores on PA but with low scores on RA. The same three clusters as described above were revealed as well as a fourth cluster of youths with a high score on PA but with a score on RA that was as high as the reactive aggression only cluster. As such, the three-cluster solution was used in all further analyses. Additional analyses showed that this three-cluster solution could be replicated in the four ethnicity groups, that the number of youths in each cluster was relatively similar across the four ethnicity groups, and that in each ethnicity group most youths were assigned to the low aggression cluster, followed by the reactive aggression only and combined aggression clusters (Table 6).

**Table 6:** Mean reactive aggression (RA) and proactive aggression (PA) scores within three cluster in the total sample and in ethnic groups

	Total (*N* = 807)			Dutch (*N* = 167)			Moroccan (*N* = 217)			Antillean/Surinamese (*N* = 160)			Mixed ethnicity (*N* = 251)		
	RA	PA	N (%)	RA	PA	N (%)	RA	PA	N (%)	RA	PA	N (%)	RA	PA	N (%)
Low aggression	4.06	.69	426 (52.8)	4.95	1.11	95 (56.9)	3.55	.42	150 (69.1)	4.74	1.08	78 (48.6)	3.99	.59	127 (50.6)
Combined aggression	13.29	9.25	102 (12.7)	14.31	9.77	13 (7.8)	11.77	8.85	17 (7.8)	13.61	9.00	20 (12.5)	12.54	9.03	34 (13.5)
Reactive aggression only	10.30	2.97	279 (34.5)	10.40	3.18	59 (27.1)	10.34	2.75	50 (23.0)	10.79	2.48	62 (38.7)	9.88	2.71	90 (35.8)

Standardized mean scores are available upon request

#### Comparing Clusters

The three clusters were not significantly different regarding the number of youths from various ethnicity, except that the low aggression cluster included more Moroccan boys (15 %) than the combined aggression cluster (13 %), but fewer than the reactive aggression only cluster (19 %). The low aggression cluster also included fewer Antillean/Surinamese boys (16 %) than the combined aggression cluster (28 %). Table 7 shows that for all variables of interest, except for peer problems, boys in the combined aggression and the reactive aggression only cluster had significantly higher scores and prevalence rates (but less prosocial behavior) than the boys in the low aggression cluster. Only boys in the reactive aggression only cluster had more peer problems than boys in the low aggression cluster. Table 7 also shows that for all variables, except depressed/anxious feelings, suicide ideation, prosocial behavior, and peer problems, boys in the combined aggression cluster had significantly higher scores and prevalence rates than boys in the reactive aggression only cluster.

**Table 7:** Means (SD) for each cluster and differences between three clusters regarding variables of interest

	Low (1)	Combined (2)	RA only (3)	Group comparison^a^		Low (1)	Combined (2)	RA only (3)	Group comparison^a^
Depressed/anxious	.76 (1.20)	1.93 (1.78)	1.57 (1.70)	**2** > 1, **3** > 1	Aggressive CD [n(%)]	11 (4.7)	29 (51.8)	23 (17.4)	2 > **1**, **3**; 3 > **1**
Suicide ideation	.09 (.43)	.41 (1.04)	.32 (.85)	**2** > 1, 3 > 1	YPI Total Score	73.01 (10.97)	106.94 (18.55)	89.46 (18.55)	2 > **1**, **3**; 3 > **1**
Social problems	1.50 (1.90)	3.91 (2.61)	2.26 (2.33)	2 > **1**, **3**; 3 > 1	YPI ID	23.56 (4.55)	35.50 (9.31)	28.29 (6.78)	2 > **1**, **3**; 3 > **1**
Prosocial behavior	8.72 (1.50)	7.58 (1.64)	8.01 (1.69)	**2** < 1, 3 < 1	YPI AD	25.13 (4.53)	34.04 (7.06)	29.33 (5.89)	2 > **1**, 3; 3 > **1**
Peer problems	1.92 (1.51)	2.32 (1.47)	2.18 (1.57)	2 > 1	YPI BD	24.29 (5.67)	37.58 (7.28)	31.90 (6.74)	2 > **1**, **3**; 3 > **1**
Aggressive behavior	1.79 (2.06)	10.12 (4.88)	5.44 (3.51)	2 > **1**, **3**; 3 > **1**	Violent Offenses	.35 (.69)	2.40 (1.51)	.84 (.96)	2 > **1**, **3**; 3 > **1**
Aggr.CD sympt.	.19 (.49)	1.18 (1.36)	.54 (.96)	2 > **1**, **3**; 3 > 1	Theft	.94 (1.51)	4.92 (2.95)	2.10 (2.11)	2 > **1**, **3**; 3 > **1**
Angry irritable	.92 (.49)	4.08 (2.11)	2.78 (2.24)	2 > **1**, **3**; 3 > **1**	Vandalism	.33 (.71)	2.60 (1.62)	.92 (1.14)	2 > **1**, **3**; 3 > **1**
Alcohol/drug use	.59 (1.28)	3.43 (2.41)	1.58 (2.04)	2 > **1**, **3**; 3 > **1**	Threats/Insults	.24 (.63)	2.25 (1.24)	0..83 (.97)	2 > **1**, **3**; 3 > **1**
SUD [n (%)]	70 (26.5)	73 (83.9)	111 (59.7)	2 > **1**, **3**; 3 > **1** ^b^	Drug-related offenses	.11 (.45)	1.19 (1.26)	.38 (.70)	2 > **1**, **3**; 3 > **1**
CD [n (%)]	14 (6.0)	35 (62.5)	27 (20.5)	2 > **1**, **3**; 3 > **1** ^b^					

*RA* reactive aggression only, *Aggr. CD sympt*. number of aggressive conduct disorder symptoms, *SUD* substance use disorder, *CD* conduct disorder, *YPI* youth psychopathic traits inventory, *ID* Interpersonal Dimension,* AD* Affective dimensions,* BD* Behavioral dimension, bold numbers refer to moderate effect sizes (Cohen’s d from .30 to .79); Underlined numbers refer to large effect sizes (Cohen’s d of .80 or higher) (for categorical variables odds ratios were calculated by a 2 × 2 table and transformed to effect size by ln (OR)/1.81 (Chin, 2000)

^a^Based on Mann–Whitney (*p* < .01) for continuous variables and Chi square (*p* < .01) for categorical variables

^b^Effect Sizes close to .80 cut-off (.78 and .77, respectively)

## Discussion

The current study was designed to test the psychometric properties of the RPQ and its usefulness to identify meaningful subgroups of detained youths. Notwithstanding that various studies already examined reactive and proactive aggression in detained youths, this study substantially contributed to the literature by examining associations with variables of interest across ethnic groups whilst using data that were gathered outside of a research context. Overall, our findings provided strong support for the factorial validity, convergent and criterion validity and the internal consistency of RPQ scores in the total sample and in each ethnicity group. In addition, three meaningful clusters of youths could be identified, with the combined RA and PA cluster including the most severe boys in terms of aggression, anger, delinquency, alcohol and drug use, psychopathic traits, and prevalence rates for CD and SUD.

Results from CFA supported the two-factor structure over the one-factor structure of the RPQ, and showed that a significant distinction can be made between reactive aggression and proactive aggression in detained adolescent males. Specifically, all model fit indices were indicative of an acceptable or good model fit in the total sample and in youths from various ethnicities, except for the *χ*^2^/*df* ratio for the total sample. However, with increasing sample size and a constant number of degrees of freedom, the *χ*^2^ value increases, and the *χ*^2^/*df* ratio, therefore, may suggest to reject a plausible model [38]. Because the *χ*^2^/*df* ratio was below the cut-off value in three subgroups and because all the other fit indices supported the two-factor model of the RPQ, this model can be considered to be acceptable in the total sample as well.

Our findings also provide support for the convergent validity of the RPQ scores. At the zero-order level, RPQ total, RA and PA scores, were positively related to other indices of aggressive behavior and features of anger-irritability. Also, after controlling for the PA score, only the RA score remained significantly related to anger and irritability, but was no longer related to aggressive CD symptoms. These results support the view that reactive, but not proactive aggression, is often accompanied with anger and a loss of impulse control [4, 5]. Although some of the aggressive CD symptoms can occur as an uncontrolled response to frustration or anger (e.g., forcing someone into sexual activity, initiating fights, using a weapon that can cause serious physical harm), the RA score was never significantly related to aggressive CD symptoms after controlling for the PA score. Yet, after controlling for the RA score, the PA score remained significantly related to aggressive CD *symptoms* (Table 3) in the total sample and Dutch and Moroccan boys. This suggests that the aggression displayed by detained youths with a CD diagnosis is likely to be premeditated and planned, a notion that is supported by the finding that only the PA score was positively related to aggressive conduct *disorder* (Tables 4, 5).

The results also supported the criterion validity of the RPQ score in detained male youths. As hypothesized, only the RA score was positively related to depressive feelings, anxiety, and suicide ideation after controlling for the other RPQ scale score. Although there were no clear expectations about the relationship between the RPQ and social problems, the RA score was positively associated with this outcome in the total sample and some ethnicity groups. Overall, our findings are in accordance with recent work, including studies that scrutinized relations with suicide risk, social problems and peer rejection [41, 42], and support the claim that reactive aggression is an indicator of overall poor psychosocial adjustment [13]. However, the results do not support the suggestion that reactive aggression is primarily related to low prosocial behavior, and that proactive aggression has little or no association with prosocial behavior independent of reactive aggression [13]. In contrast, the present study showed that only the PA score was significantly negatively related to prosocial behavior after controlling for the RA score. Given that few studies addressed the relationship between self-reported reactive and proactive aggression and prosocial behavior, future studies are warranted. The finding that a higher PA score was associated with a lower level of prosocial behavior, nevertheless, corresponds with the finding that only PA was positively related to self-reported offenses (Tables 4, 5).

After controlling for the other RPQ scale score, only the PA score was positively related to alcohol and drugs use and a SUD in boys from Dutch, Moroccan and Antillean/Surinamese ethnicity. Yet, in boys of mixed ethnicity the RA score also was positively related to both outcomes (after controlling for the RA score), which may explain why this relationship was also reported for the total sample. Overall, this study supports the suggestion that proactive aggression is more likely to be related to alcohol and substance use than reactive aggression [43], and replicates prior RPQ work with students that showed that only the PA score was positively related to substance use [7]. Also, in agreement with this latter study [7] was the finding that only the PA score was positively related to violence and theft, whereas the RA score was not. This study added to the rather limited literature on the relationship between reactive and proactive aggression and delinquency by showing that the RPQ’s PA score, but not its RA score, was positively related to all other types of offenses as well (except for threats and insults in youths from a the mixed ethnicity group). This suggests that youths with high levels of proactive aggression are not only amongst the most violent offenders, but are also likely to be versatile offenders. Finally, and after controlling for the other RPQ scale score, only the PA score was significantly positively related to the affective (and interpersonal) psychopathy dimension, a finding that supports the view that proactive aggression is a cold form of aggression [44].

Although most often small in magnitude, significant RPQ mean score differences between the four ethnicity groups were revealed, with Moroccan boys reporting the lowest RA and PA scores. In each ethnic group, boys had higher RA scores than PA scores a finding that dovetails with prior RPQ work that compared youths from various other ethnic groups [16, 24]. Of note, Moroccan boys had the lowest scores on aggression, but also on most of the other variables used in this study, including mental health problems (e.g., depressed/anxious, angry-irritable, alcohol/drug use), and self-reported offenses, a finding that also converges with prior work in boys being detained in the Netherlands [21, 45]. Importantly, the validity of the RPQ scores was well supported in all four ethnicity groups, a finding that bears substantial clinical relevance as detained youths are most often not from the major ethnicity group of the country where they live and are being detained. Thus, to the extent that clinicians have an interest in assessing reactive and proactive aggression through standardized tools, they need to have confidence that these tools provide a reliable and valid assessment of reactive and proactive aggression regardless of the boys’ ethnicity. The present study provides preliminary support that the RPQ fulfills this requirement.

Person-centered analyses identified three groups of detained youths that previously had been labeled as low aggression, combined aggression, and reactive aggression only [23]. Importantly, these groups differed from each other in a meaningful and clinically relevant way. Specifically, boys in the combined aggressive cluster had more social problems, displayed more aggression, and anger, more often met criteria for CD and SUD, reported higher levels of psychopathic traits, and committed more violent and other types of offenses than boys in the other two clusters and effect sizes (*ES*) indicated that most of these differences were large. Of note, boys in the reactive aggression only cluster were also more disturbed than boys in the low aggression cluster. These results clearly suggest that the combined aggression and reactive aggression only clusters differ in severity of risk factors rather than in the type of risk factor [22]. Of note, our finding contrasts the notion that CU traits are a unique risk factor for combined aggressive youths [22, 23, 46]. In the present study, both the combined aggression and the reactive aggression only clusters had much higher levels of CU traits than boys from the low aggression cluster (*ES* of .80 or higher), whilst the difference in the level of CU traits between both the combined aggression and reactive aggression only clusters was small in magnitude (*ES* ≤ .30). Future studies are needed, especially because it has been speculated that juveniles with high levels of reactive aggression are low in CU traits and are distressed by the effect of their behavior [22, 46]. Finally, although the combined aggression and reactive aggression only clusters were significantly different in terms of depressive and anxious feelings, and suicide ideations (see also [18]), boys in both clusters displayed higher levels of these problems than boys in the low aggression cluster. Taken together, reactive aggression is likely to be a robust risk factor for internalizing turmoil [13], whether or not co-occurring with proactive aggression.

As always, the results of this study must be interpreted in the context of several limitations. First, the cross-sectional study design does not allow making conclusions about the temporal relationships between reactive and proactive aggression and criterion variables of interest. Second, due to the sole reliance on self-reported information, it cannot be excluded that strong associations between variables of interest and differences between the clusters are inflated due to shared method variance. Third, the number of youths within each of the ethnic groups was too restricted to determine whether the differences and similarities between the three clusters could have been replicated in each of these groups. Fourth, no experimental design was used to determine whether RPQ scores differed between youths who had or had not been assured that the information they provided would be handled confidentially and anonymously. Fifth, only detained males were included in this study, implicating that this is the only population that an inference can be drawn upon.

### Summary

This study suggests that the RPQ provides a reliable and valid assessment of reactive and proactive aggression in severely antisocial, criminal-justice involved adolescents from different ethnicities, even in a context in which confidentiality and anonymity of the information are not guaranteed. Both the variable-oriented and person-oriented analyses supported the notion that there are meaningful differences between reactive and proactive aggression, with reactive aggression being most robustly related to internalizing problems and disorders, and proactive aggression being most robustly related to externalizing problems and disorders, self-reported offending and psychopathic-traits. These findings altogether support prior suggestions that reactively and proactively aggressive youths have different treatment needs [47].

## Electronic supplementary material

Supplementary material 1 (DOCX 14 kb)

## Footnotes

1 The MAYSI-2 was specifically designed for use in juvenile justice involved youths. Therefore, if constructs that are relevant (e.g., depressive feelings) to test the convergent and criterion validity of the RPQ were also measured through questionnaires other than the MAYSI-2, the MAYSI-2 was given preference.

2 For each ethnicity group, descriptive information for variables other than the RPQ scores is presented in a Supplementary Table.
