# Supplementary material for: Assessing Reactive and Proactive Aggression in Detained Adolescents Outside of a Research Context
Source: Child Psychiatry Hum Dev. 2015 Jun 2;47:159–72. doi: 10.1007/s10578-015-0553-z (PMC4712219; doi:10.1007/s10578-015-0553-z)
Supplement: Supplementary file 1 — Supplementary material 1 (DOCX 14 kb) [file 10578_2015_553_MOESM1_ESM.docx]

| Supplementary Material  Descriptive Information Across Ethnicity Groups and Between-group Comparisons | | | | | | | | | |
| --- | --- | --- | --- | --- | --- | --- | --- | --- | --- |
|  | Dutch (1) | | Moroccan (2) | | Antil/Surin (3) | | Mixed (4) | | Group Comparison ^a^ |
|  | *M* | *SD* | *M* | *SD* | *M* | *SD* | *M* | *SD* |  |
| Age | 16.95 | 1.42 | 16.71 | 1.06 | 16.53 | 1.30 | 16.65 | 1.40 |  |
| Depresses/Anxious | 1.47 | 1.53 | 0.90 | 1.49 | 1.27 | 1.51 | 1.16 | 1.54 | 2<1 |
| Suicide Ideation* | 0.37 | 0.89 | 0.10 | 0.52 | 0.23 | 0.68 | 0.19 | 0.69 | 2<1,3; 4<1 |
| Social Problems* | 2.87 | 2.72 | 1.42 | 1.66 | 2.44 | 2.34 | 2.09 | 2.29 | 2<1,3 |
| Prosocial Behavior | 8.01 | 1.68 | 8.59 | 1.75 | 8.23 | 1.53 | 8.40 | 1.53 | 2>1 |
| Peer Problems | 2.11 | 1.67 | 1.87 | 1.46 | 2.17 | 1.54 | 2.10 | 1.49 |  |
| Aggressive Behavior* | 5.50 | 4.99 | 2.66 | 3.38 | 4.88 | 4.37 | 3.66 | 3.67 | 2<1,3; 4<1 |
| Number CD Symptoms* | 0.60 | 1.05 | 0.19 | 0.58 | 0.62 | 0.98 | 0.38 | 0.84 | 2<1,3 |
| Angry Irritable* | 2.77 | 2.49 | 1.37 | 1.98 | 2.26 | 2.13 | 1.75 | 1.93 | 2<1,3,4; 4<1 |
| Alcohol/Drug use* | 2.47 | 2.38 | 0.50 | 1.29 | 1.23 | 1.65 | 1.25 | 2.00 | 2<1,3,4; 3,4<1 |
| SUD [N(%)] | 90 | 65.2 | 29 | 22.7 | 58 | 51.8 | 75 | 49.7 | 2<1,3,4; 1>4 |
| CD [N(%)] | 25 | 25.0 | 13 | 11.5 | 20 | 22.2 | 17 | 15.2 |  |
| Aggressive CD [N(%)] | 20 | 20.0 | 9 | 8.0 | 17 | 18.9 | 16 | 14.3 |  |
| YPI Total Score | 87.81 | 18.46 | 78.52 | 17.57 | 85.92 | 16.64 | 82.11 | 17.05 | 2<1,3 |
| YPI Interpersonal | 27.75 | 7.94 | 25.44 | 7.08 | 27.98 | 7.18 | 26.36 | 6.96 | 2<3 |
| YPI Affective | 28.24 | 6.62 | 26.88 | 6.06 | 28.63 | 6.23 | 27.60 | 6.04 |  |
| YPI Behavioral | 31.85 | 8.22 | 26.20 | 7.61 | 29.48 | 7.23 | 28.00 | 7.72 | 2<1,3; 1>4 |
| Violent Offenses* | 1.17 | 1.37 | 0.41 | 0.78 | 1.04 | 1.28 | 0.55 | 0.92 | 2<1,3; 1,3>4 |
| Theft* | 2.61 | 2.91 | 1.25 | 1.53 | 1.86 | 2.24 | 1.62 | 2.09 | 2<1, |
| Vandalism* | 1.29 | 1.53 | 0.44 | 0.85 | 0.99 | 1.28 | 0.67 | 1.22 | 2<1;3 1>4 |
| Threats/Insults* | 0.66 | 0.99 | 0.15 | 0.53 | 0.40 | 0.83 | 0.28 | 0.70 | 2<1,3; 1>4 |
| Drug-related offenses* | 0.37 | 0.52 | 0.17 | 0.35 | 0.35 | 0.48 | 0.28 | 0.49 | 2<1,3 |
| *Note.* CD = Conduct Disorder *;* SUD = Substance Use Disorder; YPI = Youth Psychopathic traits Inventory;  ^a^ Based on one-way anova for continuous variables and Chi-Square (p <.01) for categorical variables; * Mann-Whitney (p < .01) instead of one-way anova | | | | | | | | | |
